# Ranking Medical Terms to Support Expansion of Lay Language Resources for Patient Comprehension of Electronic Health Record Notes: Adapted Distant Supervision Approach

**DOI:** 10.2196/medinform.8531

**Published:** 2017-10-31

**Authors:** Jinying Chen, Abhyuday N Jagannatha, Samah J Fodeh, Hong Yu

**Affiliations:** ^1^ Department of Quantitative Health Sicences University of Massachusetts Medical School Worcester, MA United States; ^2^ School of Computer Science University of Massachusetts Amherst, MA United States; ^3^ Yale Center for Medical Informatics Yale University New Haven, CT United States; ^4^ Bedford Veterans Affairs Medical Center Bedford, MA United States

**Keywords:** electronic health records, natural language processing, lexical entry selection, transfer learning, information extraction

## Abstract

**Background:**

Medical terms are a major obstacle for patients to comprehend their electronic health record (EHR) notes. Clinical natural language processing (NLP) systems that link EHR terms to lay terms or definitions allow patients to easily access helpful information when reading through their EHR notes, and have shown to improve patient EHR comprehension. However, high-quality lay language resources for EHR terms are very limited in the public domain. Because expanding and curating such a resource is a costly process, it is beneficial and even necessary to identify terms important for patient EHR comprehension first.

**Objective:**

We aimed to develop an NLP system, called adapted distant supervision (ADS), to rank candidate terms mined from EHR corpora. We will give EHR terms ranked as high by ADS a higher priority for lay language annotation—that is, creating lay definitions for these terms.

**Methods:**

Adapted distant supervision uses distant supervision from consumer health vocabulary and transfer learning to adapt itself to solve the problem of ranking EHR terms in the target domain. We investigated 2 state-of-the-art transfer learning algorithms (ie, feature space augmentation and supervised distant supervision) and designed 5 types of learning features, including distributed word representations learned from large EHR data for ADS. For evaluating ADS, we asked domain experts to annotate 6038 candidate terms as important or nonimportant for EHR comprehension. We then randomly divided these data into the target-domain training data (1000 examples) and the evaluation data (5038 examples). We compared ADS with 2 strong baselines, including standard supervised learning, on the evaluation data.

**Results:**

The ADS system using feature space augmentation achieved the best average precision, 0.850, on the evaluation set when using 1000 target-domain training examples. The ADS system using supervised distant supervision achieved the best average precision, 0.819, on the evaluation set when using only 100 target-domain training examples. The 2 ADS systems both performed significantly better than the baseline systems (*P*<.001 for all measures and all conditions). Using a rich set of learning features contributed to ADS’s performance substantially.

**Conclusions:**

ADS can effectively rank terms mined from EHRs. Transfer learning improved ADS’s performance even with a small number of target-domain training examples. EHR terms prioritized by ADS were used to expand a lay language resource that supports patient EHR comprehension. The top 10,000 EHR terms ranked by ADS are available upon request.

## Introduction

### Significance and Background

Online patient portals have been widely adopted in the United States in a nationwide effort to promote patient-centered care [1-3]. Many health organizations also allow patients to access their full electronic health record (EHR) notes through patient portals, with early evidence showing improved medical comprehension and health care outcomes [4-6]. However, medical terms—abundant in EHR notes—remain a major obstacle for patients to comprehend medical text, including EHRs [7-12]. In addition, an estimated 36% of adult Americans have limited health literacy [13]. Limited health literacy has been identified as one major barrier to patient use of EHRs [3,14-17]. Misinterpretation of EHR content may result in unintended increases in service utilization and change of patient-provider relationships.

Textbox 1 shows an excerpt from a typical clinical note. The medical terms that may hinder patients’ comprehension are italicized. Here we show a subset of medical terms identified by the Unified Medical Language System (UMLS) lexical tool MetaMap [18] for illustration purposes only.

**Illustration of medical terms in a sample clinical note.**

Her *creatinine* has shown a steady rise over the past four years. She does have *nephrotic range proteinuria*. The likely *etiology* of her *nephrotic range proteinuria* is her *diabetes*.

She was on an *ACE inhibitor*, which was just stopped in August due to the *elevated creatinine* of 4.41. Given the severity of her *nephrotic syndrome*, her chronic kidney disease is likely permanent; however, I will repeat a *chem-8* now that she is off the *ACE inhibitor*. I will also get a *renal duplex scan* to make sure she does not have any *renal artery stenosis*.

There has been long-standing research interest in developing health information technologies that promote health literacy and consumer-centered communication of health information [19,20]. Natural language processing (NLP)-enabled interventions have also been developed to link medical terms in EHRs to lay terms [21,22] or definitions [23], showing improved comprehension [22,23]. Although there is a substantial amount of health information available on the Internet, many Internet users face challenges accessing and selecting relevant high-quality information [24-27]. The aforementioned NLP-enabled interventions have the advantage of reducing patients’ information-seeking burden by integrating authorized health-related information in a single place, and thereby helping patients easily read through and understand their EHR notes.

However, high-quality lay language resources—the cornerstone of such interventions—are very limited in the public domain. The readability levels of health educational materials on the Internet often exceed the level that is easily understood by the average patient [28-30]. Definitions of medical terms provided by controlled health vocabularies, such as those included in the UMLS, often themselves contain complex medical concepts. For example, the term “nephrotic syndrome” in Textbox 1 is defined in the US National Cancer Institute vocabulary as “A collection of symptoms that include severe edema, proteinuria, and hypoalbuminemia; it is indicative of renal dysfunction,” where the medical concepts “edema,” “proteinuria,” “hypoalbuminemia,” and “renal dysfunction” may not be familiar to patients.

The consumer health vocabulary (CHV) [31] is a valuable lay language resource that has been integrated into the UMLS and has also been used in EHR simplification [21,22]. CHV contains consumer health terms (which were used by lay people to query online health information) and maps these terms to UMLS concepts. As a result, it contains both lay terms and medical terms, and links between these 2 types of terms. In addition, it provides lay definitions for some medical terms. From our current work, however, we found that CHV alone is not sufficient for comprehending EHR notes, as many medical terms in EHRs do not exist in CHV, and many others exist in CHV but do not have lay terms or lay definitions. For example, among the 19,503 unique terms identified by MetaMap [18] from a corpus of 7839 EHR notes, 4680 (24.0%) terms do not appear in CHV, including “focal motor deficit,” “Hartmann procedure,” “titrate,” and “urethrorectal fistula” (see Multimedia Appendix 1 for more results).

We are building a lay language resource for EHR comprehension by including medical terms from EHRs and creating lay definitions for those terms. This is a time-consuming process that involves collecting candidate definitions from authorized health educational resources, and curating and simplifying these definitions by domain experts. Since the number of candidate terms mined from EHRs is large (hundreds of thousands of terms), we ranked candidate terms based on how important they are for patients’ comprehension of EHRs, and therefore prioritized the annotation effort of lexical entries based on those important terms.

The goal of this study was to develop an NLP system to automate the process of lexical entry selection. This task was challenging because the distinctions between important and nonimportant EHR terms in our task were more subtle than that between medical terms and nonmedical terms (detailed below in the Important Terms for Electronic Health Record Comprehension subsection). To achieve this goal, we developed a new NLP system, called adapted distant supervision (ADS), which uses distant supervision from the CHV and uses transfer learning to adapt itself to the target domain to rank terms from EHRs. We aimed to empirically show that ADS is effective in ranking EHR terms at the corpus level and outperforms supervised learning.

### Related Work

#### Natural Language Processing to Facilitate Creation of Lexical Entries

Previous studies have used both unsupervised and supervised learning methods to prioritize terms for inclusion in biomedical and health knowledge resources [32-35]. Term recognition methods, which are widely used unsupervised methods for term extraction, use rules and statistics (eg, corpus-level word and term frequencies) to prioritize technical terms from domain-specific text corpora. Since these methods do not use manually annotated training data, they have better domain portability but are less accurate than supervised learning [32]. The contribution of this study is to propose a new learning-based method for EHR term prioritization, which is more accurate than supervised learning while also having good domain portability.

Our work is also related to previous studies that have used distributional semantics for lexicon expansion [35-37]. In this work, we used word embedding, one technique for distributional semantics, to generate one type of learning features for the ADS system to rank EHR terms.

#### Ranking Terms in Electronic Health Records

We previously developed NLP systems to rank and identify important terms from each EHR note of individual patients [38,39]. This study is different in that it aimed to rank terms at the EHR corpus level for the purpose of expanding a lay language resource to improve health literacy and EHR comprehension of the general patient population. Notice that both types of work are important for building NLP-enabled interventions to support patient EHR comprehension. For example, a real-world application can link all medical jargon terms in a patient’s EHR note to lay terms or definitions, and then highlight the terms most important for this patient and provide detailed information for these important terms.

#### Distant Supervision

Our ADS system uses distant supervision from the CHV. Distant supervision refers to the learning framework that uses information from knowledge bases to create labeled data to train machine learning models [40-42]. Previous work often used this technique to address context-based classification problems such as named entity detection and relation detection. In contrast, we used it to rank terms without considering context. However, our work is similar in that it uses heuristic rules and knowledge bases to create training data. Although training data created this way often contain noise, distant supervision has been successfully applied to several biomedical NLP tasks to reduce human annotation efforts, including extraction of entities [40,41,43], relations [44-46], and important sentences [47] from the biomedical literature. In this study, we made novel use of the non-EHR-centric lexical resource CHV to create training data for ranking terms from EHRs. This approach has greater domain portability than conventional distant supervision methods due to fewer demands on the likeness between the knowledge base and the target-domain learning task. On the other hand, learning from the distantly labeled data with a mismatch to the target task is more challenging. We address this challenge by using transfer learning.

#### Transfer Learning

Transfer learning is a learning framework that transfers knowledge from the source domain *D*_S_ (the training data derived from the CHV, in our case) to the target domain *D*_T_ to help improve the learning of the target-domain task *T*_T_ [48]. We followed Pan and Yang [48] to distinguish between inductive transfer learning, where the source- and target-domain tasks are different, and domain adaptation, where the source- and target-domain tasks are the same but the source and target domains (ie, data distributions) are different. Our approach belongs to the first category because our source-domain and target-domain tasks define positive and negative examples in different ways. Transfer learning has been applied to important bioinformatics tasks such as DNA sequence analysis and gene interaction network analysis [49]. It has also been applied to several clinical and biomedical NLP tasks, including part-of-speech tagging [50] and key concept identification for clinical text [51], semantic role labeling for biomedical articles [52] and clinical text [53], and key sentence extraction from biomedical literature [47]. In this work, we investigated 2 state-of-the-art transfer learning algorithms that have shown superior performance in recent studies [47,53]. We aimed to empirically show that they, in combination with distant supervision, are effective in ranking EHR terms.

## Methods

### Electronic Health Record Corpus and Candidate Terms

**Figure 1:**
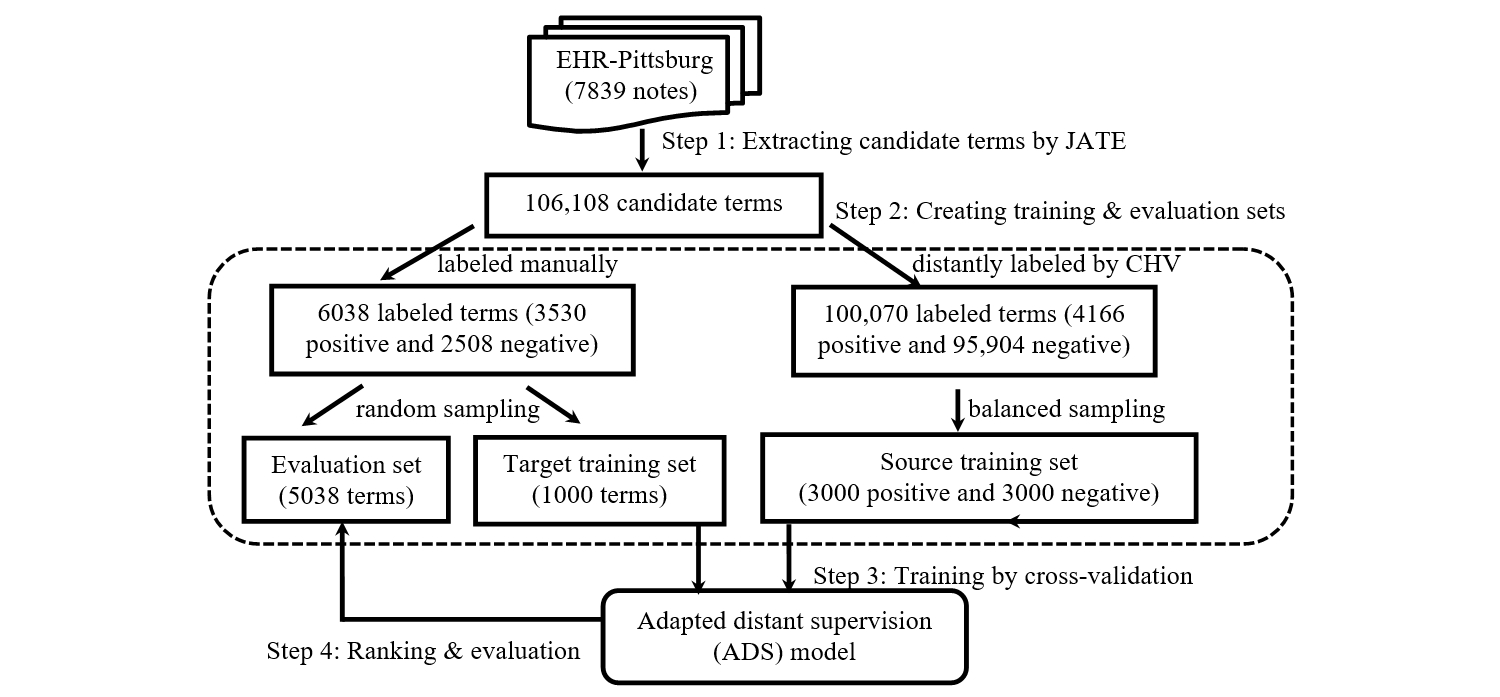
Overview of development of the adapted distant supervision (ADS) natural language processing system to rank candidate terms mined from electronic health record (EHR) corpora: data extraction (steps 1 and 2), ADS (step 3), and evaluation (step 4). CHV: consumer health vocabulary.

We used 7839 discharge summary notes (5.4 million words) from the University of Pittsburgh NLP Repository (using these data requires a license) [54], called EHR-Pittsburgh for convenience, for this study. We applied the linguistic filter of the Java Automatic Term Extraction (JATE) toolkit (version 1.11) [55] to EHR-Pittsburgh to extract candidate terms (see step 1 in Figure 1). JATE’s linguistic filter uses a word extractor, a noun phrase extractor, and a stop word list to select high-quality words and noun phrases as candidate terms. We extracted a total of 106,108 candidate terms and further used them to identify and rank medical terms.

### Consumer Health Vocabulary

CHV was developed by collaborative research to address vocabulary discrepancies between lay people and health care professionals [56-59]. CHV incorporates terms extracted from various consumer health sites, including queries submitted to MedlinePlus, a consumer-oriented online knowledge resource maintained by the US National Library of Medicine [60,61]. CHV contains 152,338 terms, most of which are consumer health terms [60-62]. Zeng et al [60] mapped these terms to UMLS concepts by a semiautomatic approach. As a result of this work, CHV encompasses lay terms (eg, “low blood sugar level” and “heart attack”), as well as corresponding medical terms (eg, “hypoglycemia” and “myocardial infarction”). In this study, we used CHV to create distantly labeled training data for ADS.

### Important Terms for Electronic Health Record Comprehension

We defined important terms as those terms that, if understood by the patients, would significantly improve their EHR comprehension. In practice, we used 4 criteria, unithood, termhood, unfamiliarity, and quality of compound term (defined with examples in Multimedia Appendix 2), to judge term importance.

Except for unithood, which is a general criterion for lexical entry selection, the other 3 criteria all measure term importance from the perspective of patient EHR comprehension (details in Multimedia Appendix 2). For example, familiar terms are not important because they are already known by the average patient. High-quality compound terms are those terms whose meanings are beyond the simple sum of their component words (eg, “community-acquired pneumonia”). These terms are important and should be annotated with lay definitions because otherwise patients would not understand them even if they know all the individual words in these terms.

### Distant Supervision from Consumer Health Vocabulary

We used CHV to select positive examples to train ADS (see step 2 in Figure 1). Specifically, we assumed that medical terms that occur in both EHRs and CHV (called EHR-CHV terms) are important for patient EHR comprehension. We chose CHV for distant supervision for 3 reasons. First, terms in CHV have been curated and thus all satisfy the unithood criterion. Second, recall that medical terms existing in CHV are synonyms of consumer health terms initially identified from queries and postings generated by patients in online health forums. Therefore, we expect most of these terms to bear clear and significant clinical meanings for patients and thus satisfy the termhood criterion. Third, CHV assigns familiarity scores to 57.89% (88,189 out of 152,338) of its terms for extended usability, which can be used to distinguish between medical terms and lay terms. CHV familiarity scores estimate the likelihood that a term can be understood by an average reader [63] and take values between 0 and 1 (with 1 being most familiar and 0 being least familiar). CHV provides different types of familiarity scores [21]. Following Zeng-Treitler et al [21], we used the combined score and used a heuristic rule (ie, CHV familiarity score ≤0.6) to identify medical terms.

Despite the aforementioned merits, CHV is not perfect in labeling the training data. First, there is not a clear boundary between familiar and unfamiliar terms if their CHV familiarity scores are close to 0.6. For example, “congestive heart failure” and “atypical migraine” have familiarity scores of 0.64 and 0.61; therefore, they would be labeled as negative examples by CHV. However, these 2 terms were judged by domain experts as important terms that need lay definitions. Second, some compound terms in CHV (eg, “knee osteoarthritis,” “brain MRI,” “aspirin allergy”), although labeled as positive examples by CHV, were judged by domain experts as being not high-quality compound terms from the perspective of efficiently expanding a lay language resource and thus did not need immediate treatment for adding lay definitions.

### Transfer Learning Algorithms

#### Problem Formalization

Since CHV-labeled training data are noisy, we used transfer learning to adapt the system distantly supervised by CHV to the target-domain task. More formally, we defined the training data derived from CHV as the source-domain data *D_S_*={(*x^s^*_1_*, y^s^*_1_), (*x^s^*_2_*, y^s^*_2_), …, (*x^s^_N_, y^s^_N_*)} and the target-domain data as *D_T_*={(*x^t^*_1_*, y^t^*_1_), (*x^t^*_2_*, y^t^*_2_), …, (*x^t^_M_, y^t^_M_*)}, where *N* is the number of source-domain instances, (*x^s^, y^s^*) is the paired feature vector and class label of an instance in the source domain, and *M* and (*x^t^, y^t^*) are defined similarly for the target domain. Notice that we refer to CHV-labeled candidate terms as the source-domain data by following the convention of transfer learning, although these terms were extracted from EHRs. In our study, we used all the *N* source-domain instances and at most *K* (*K* « *M*) target-domain instances to train the model. The goal of transfer learning is to make an optimal use of the *N+K* training data to improve model performance on the *M-K* target-domain test data.

In this study, we investigated 2 state-of-the-art transfer learning methods: feature space augmentation (FSA) and supervised distant supervision (SDS).

#### Feature Space Augmentation

FSA [64] has shown the best performance in semantic role labeling for clinical text [53].

**Figure 2:**
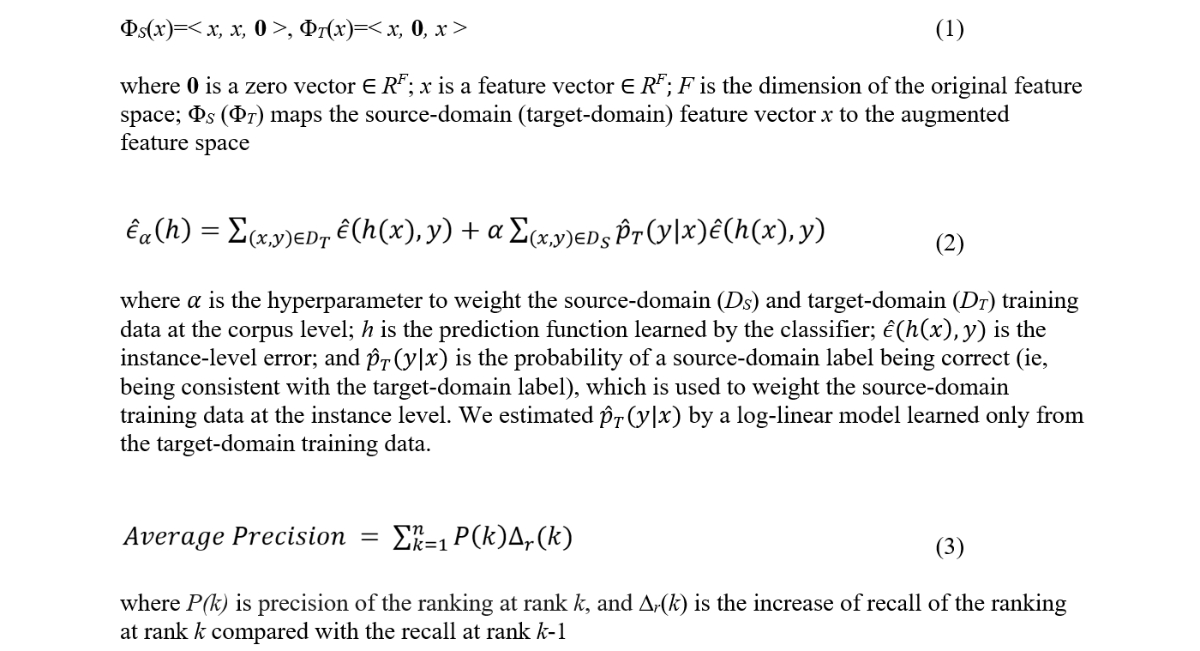
Equations for feature mapping functions used in feature space augmentation (1), objective function used in supervised distant supervision (2), and average precision (3).

This approach assumes that *D_S_* and *D_T_* share the same feature space *X*= *R^F^* (ie, each feature vector is an *F*-dimension real-valued vector) and defines an augmented feature space *X*^+^= *R^3F^*. It then defines 2 feature mapping functions, Φ*_S_* and Φ*_T_*: *X→* *X*^+^, by Equation 1 (Figure 2) to respectively map feature vectors from *D_S_* and *D_T_* to the augmented feature space. The motivation is to make the learning easier by separating the general features (ie, the first *F* dimensions in the augmented feature space, which are useful to learn examples in both *D_S_* and *D_T_*) and the domain-specific features (the second and third *F* dimensions in the augmented feature space). In addition, it allows a single model to regulate jointly the trade-off between the general and domain-specific feature weights.

#### Supervised Distant Supervision

SDS is an extension of the algorithm recently proposed by Wallace et al [47]. It minimizes an objective function that combines empirical source-domain and target-domain errors, as defined in Equation 2 (Figure 2).

Our algorithm differs from that of Wallace et al [47] in that it does not assume that only positive examples in the source domain are unreliable and is therefore more generalizable.

#### Implementation Issues

We implemented 2 versions of the ADS system, ADS-fsa and ADS-sds, by incorporating the 2 transfer learning algorithms. We used the log-linear model as the base of all the models (including the baseline models introduced in the subsection Baseline Systems) and used L2 regularization for model training. The output from the log-linear models is probabilities of a candidate term being a positive example and can be used to rank candidate terms directly. We used grid search and cross-validation on the target-domain training data to set the hyperparameters *α* (the corpus weighting parameter in Equation 2; Figure 2) and *C* (the hyperparameter of the log-linear model to control the regularization strength; a small *C* corresponds to a strong regularization). In our experiments, we set *α*=*β*(*K* / *N*) (*N* and *K* are the size of the source- and target-domain training data) and searched *β* in [0.01, 0.1, 1, 10, 100]. We searched *C* in [1,0.1,0.001,0.0001].

### Training and Evaluation Datasets

#### Data Annotation

We derived the training and evaluation datasets from the 106,108 candidate terms extracted from EHR-Pittsburgh as follows.

First, 3 people with a postgraduate level of education in biology, public health, and biomedical informatics reviewed candidate terms among the terms ranked as high by the nonadapted distant supervision model (ie, among the top 10,000 terms) or by the term recognition algorithm C-value [65] (ie, among the top 5000 terms). We chose top-ranked terms, which were likely to contain more important terms than randomly sampled terms, to speed up the whole annotation process. We used the output from 2 methods to increase the diversity of terms used for evaluation and used more terms from the distant supervision model because a manual review suggested that it outperformed C-value. We adopted the expert annotation approach because nonexperts may lack sufficient knowledge to judge the domain relevance and quality of a candidate term, which could potentially introduce noise to the data and slow down the annotation process.

Each term was annotated by 1 primary reviewer and then reviewed by another reviewer based on the 4 criteria introduced in the subsection Important Terms for Electronic Health Record Comprehension (details in Multimedia Appendix 2). Difficult cases were discussed and resolved within the group. Using this procedure, we obtained 6038 annotated terms (3530 positive examples and 2508 negative examples) before starting this study and used all of them for our experiments. To compute the interannotator agreement, 2 reviewers independently annotated 500 candidate terms and achieved a .71 kappa coefficient on this dataset.

#### Target-Domain Training and Evaluation Sets

We used 1000 examples randomly sampled from the 6038 annotated terms as the target-domain training set and used the remaining 5038 terms as the evaluation set. We did not use stratified sampling because in practice we did not know the class distribution of the target-domain data or the test data. In transfer learning, the target-domain training data are critical to system performance. Therefore, we repeated the above procedure 100 times to obtain 100 pairs of <target training set, evaluation set> for system evaluation to take into account the variance of the target training set. To test the effects of the size of the target-domain training data, we reported system performance by using *L* (*L*=100, 200, 500, 1000) examples randomly selected from the full target training set.

#### Source-Domain Training Set

We first obtained 100,070 terms by removing the 6038 manually labeled terms from the 106,108 candidate terms. We then automatically labeled the 100,070 terms based on whether a term was an EHR-CHV medical term (ie, positive term) or not (ie, negative term). In this way, we obtained 4166 positive terms and 95,904 negative terms. Because we did not know the distribution of the target-domain data, we randomly sampled 3000 positive and 3000 negative terms from these data to form a balanced source-domain training set. We set the size of the source training set to 6000 by following previous work [66].

### Baseline Systems

We employed 2 baselines commonly used to evaluate transfer learning methods [47,53,64]: *SourceOnly* or nonadapted distant supervision model, which was trained by using only source-domain training data, and *TargetOnly*, which was trained by using only target-domain training data.

### Features

#### Word Embedding

Word embedding is the distributed vector representation of words. It has emerged as a powerful technique for word representation and proved beneficial in a variety of biomedical and clinical NLP tasks. We used word2vec software to create the skip-gram word embeddings [67,68] and trained word2vec using a combined text corpus (over 3 billion words) of English Wikipedia, articles from PubMed Central Open Access Subset, and 99,735 EHR notes from the University of Pittsburgh NLP Repository [54]. We set the training parameters by following Jagannatha et al [37] and Pyysalo et al [69]. Specifically, we used 200-dimension vectors with a window size of 6 and used hierarchical soft-max with a subsampling threshold of 0.001 for training. We represented multiword terms (ie, compound terms) by the mean of the vectors of their component words by following Jagannatha et al [37] and Chen and colleagues [38,39].

#### Semantic Type

We mapped candidate terms to UMLS concepts and included semantic types for those concepts that had an exact match or a head-noun match as features. Each semantic type is a 0-1 binary feature. This type of feature has been used to identify domain-specific medical terms [23,33] and to rank medical terms from individual EHR notes [38].

#### Automatic Term Recognition

We used the confidence scores from 2 term-recognition algorithms: corpus-level term frequency-inverse document frequency [55] and C-value [65].

#### General-Domain Term Frequency

We generated 4 features from the Google Ngram corpus [70]: the average, minimum, and maximum frequencies of a term’s component words and the term frequency. Corpus frequency has proved to be a strong indicator for term familiarity [63,71]. The Google Ngram corpus is a database of unigram and n-gram counts of words collected from over 15 million books containing over 5 billion pages. We used the top 4.4 million high-frequency words from this corpus and their unigram, bigram, and trigram matches to derive our features.

#### Term Length

Term length is the number of words in a term. Because a long candidate term may not be a good compound term but rather a simple concatenation of shorter terms (eg, “left heart cardiac catheterization”), this feature may help the ADS system to identify and rank as low the low-quality compound terms.

### Evaluation Metrics

#### Average Precision

This metric averages precision *P(k)* at rank *k* as a function of recall *r*, as defined in Equation 3 (Figure 2).

#### Area Under the Receiver Operating Characteristic Curve

The area under the receiver operating characteristic curve (AUC-ROC) is computed; this curve plots the true positive rate (y-coordinate) against the false positive rate (x-coordinate) at various threshold settings.

Recall that we have 100 pairs of <target training set, evaluation set> randomly sampled from the 6038 labeled terms. When evaluating a system, we averaged its performance scores on the 100 pairs of datasets and report the averaged values.

We used sklearn.metrics to compute the average precision and AUC-ROC scores. Scikit-learn is an open source Python library widely used for machine learning [72]. In this study, we only reported the paired-samples *t* test results for performance difference between the ADS systems and the baselines because the baselines were expected to be better than a random classifier. The AUC-ROC score of each individual system tested in our experiments was significantly better than 0.5—that is, the AUC-ROC score of a random classifier (*P*<.001).

### Statistical Analysis

We used the paired-samples *t* test to test the significance of the performance difference between a pair of systems. We used scipy.stats to conduct the paired *t* test. SciPy is an open source Python library widely used for scientific computing [73].

## Results

### ADS Ranking Performance on Evaluation Set

Table 1 shows the evaluation results, where the 2 ADS systems outperformed the 2 baselines significantly (*t*_99_ ranges from 4.84 to 133.31, *P*<.001) for AUC-ROC and average precision under all 4 conditions (ie, using 4 different sizes of target training data). The performance scores of the ADS systems continuously improved with increased size of target training data. When comparing the 2 ADS systems, ADS-fsa performed significantly better than ADS-sds when using 1000 target-domain training examples for transfer learning and performed worse than ADS-sds when using 100 or 200 target-domain training examples (see bottom 2 rows in Table 1 for *t* and *P* values).

**Table 1:** Performance of different natural language processing systems on the evaluation set under 4 conditions using 100, 200, 500, and 1000 target-domain training examples^a^.

System		AUC-ROC^b^				Average precision			
		100	200	500	1000	100	200	500	1000
SourceOnly		0.739	0.739	0.739	0.739	0.811	0.811	0.811	0.811
TargetOnly		0.728	0.749	0.769	0.782	0.799	0.816	0.833	0.844
ADS-fsa^c^		0.746	0.756	*0.776*	*0.790*	0.815	0.823	*0.839*	*0.850*
ADS-sds^d^		*0.751*	*0.759*	0.775	0.786	*0.819*	*0.826*	0.838	0.847
**ADS-fsa vs ADS-sds^e^**									
	*t*_99_	4.25	2.79		8.78	3.81	3.04		11.58
	*P* values	<.001	.01		<.001	<.001	.003		<.001

^a^The highest performance scores are italicized.

^b^AUC-ROC: area under the receiver operating characteristic curve.

^c^ADS-fsa: adapted distant supervision-feature space augmentation.

^d^ADS-sds: adapted distant supervision-supervised distant supervision.

^e^The *P* values for difference between ADS-fsa and SourceOnly, ADS-sds and SourceOnly, ADS-fsa and TargetOnly, and ADS-sds and TargetOnly are <.001 (*t*_99_ ranges from 4.84 to 133.31) for all metrics under all conditions. We report the *P* values (if the *P* value ≤.05) and the corresponding *t*_99_ values for difference between ADS-fsa and ADS-sds.

The average familiarity level or score of top-ranked terms measures one important aspect of ranking quality. However, because many terms in the evaluation set did not have CHV familiarity scores, we could not compute this value directly. A manual review of the top 500 terms ranked by the best system—that is, ADS-fsa trained using 1000 target-domain training examples—did find many unfamiliar medical terms, including “autoimmune enteropathy,” “ileostomy,” “myasthenia gravis,” “nifedipine,” “parathyroid hormone,” and “phototherapy.”

### Effects of Individual Features on ADS Ranking Performance

In addition to evaluating system performance, we tested the contribution of each individual feature to system performance by using feature ablation experiments. Table 2 shows that ADS-sds’s performance dropped significantly (*P*<.001 for both measures under all 4 conditions) when respectively dropping word embedding, general-domain term frequency, and term length. Dropping the semantic features had mixed results, slightly decreasing performance when the target-domain training set was large and increasing performance when the target-domain training set was small. Dropping features derived from automatic term recognition had no statistically significant effects. The effects of dropping individual features on ADS-fsa’s performance were similar (see the first table in Multimedia Appendix 3).

**Table 2:** Performance of different ADS-sds^a^ systems implemented by using all types of features or by dropping each individual type of feature, under 4 conditions using 100, 200, 500, and 1000 target-domain training examples^b^.

ADS-sds system		AUC-ROC^c^				Average precision			
		100	200	500	1000	100	200	500	1000
ADS-sds-ALL^d^		0.751	0.759	0.775	0.786	0.819	0.826	0.838	0.847
ADS-sds-woWE^e^		0.711	0.718	0.726	0.733	0.780	0.785	0.793	0.799
**ADS-sds-woWE vs ADS-sds-ALL**									
	*t*_99_	30.37	32.74	59.92	112.25	36.61	39.63	81.04	124.15
	*P* value	<.001	<.001	<.001	<.001	<.001	<.001	<.001	<.001
ADS-sds-woSem^f^		0.753	0.760	0.772	0.782	0.823	0.829	0.838	0.845
**ADS-sds-woSem vs ADS-sds-ALL**									
	*t*_99_			4.63	12.28	3.18	4.00		4.55
	*P* value			<.001	<.001	.002	<.001		<.001
ADS-sds-woATR^g^		0.751	0.759	0.774	0.786	0.819	0.826	0.838	0.847
ADS-sds-woGTF^h^		0.740	0.749	0.765	0.777	0.813	0.821	0.833	0.842
**ADS-sds-woGTF vs ADS-sds-ALL**									
	*t*_99_	13.04	9.50	14.85	22.55	8.12	6.49	11.52	23.07
	*P* value	<.001	<.001	<.001	<.001	<.001	<.001	<.001	<.001
ADS-sds-woTL^i^		0.741	0.751	0.767	0.778	0.807	0.815	0.829	0.838
**ADS-sds-woTL vs ADS-sds-ALL**									
	*t*_99_	11.21	10.81	19.78	25.58	16.43	17.15	34.50	41.72
	*P* value	<.001	<.001	<.001	<.001	<.001	<.001	<.001	<.001

^a^ADS-sds: adapted distant supervision-supervised distant supervision.

^b^We report the *P* values (if the *P* value ≤.05) and the corresponding *t*_99_ values for differences between each implementation and ADS-sds-ALL.

^c^AUC-ROC: area under the receiver operating characteristic curve.

^d^ADS-sds-ALL: ADS-sds with all types of features.

^e^ADS-sds-woWE: ADS-sds without word embedding.

^f^ADS-sds-woSem: ADS-sds without semantic features.

^g^ADS-sds-woATR: ADS-sds without features derived from automatic term recognition.

^h^ADS-sds-woGTF: ADS-sds without general-domain term frequency.

^i^ADS-sds-woTL: ADS-sds without term length.

## Discussion

### Principal Results

In an effort to build a lexical resource that provides lay definitions for medical terms in EHRs, we developed the ADS system to rank candidate terms mined from an EHR corpus and prioritized our efforts to collect and curate lay definitions for top-ranked terms. Given only 100 labeled target training examples, the best ADS system, ADS-sds, achieved 0.751 AUC-ROC and 0.819 average precision on the evaluation set, which are significantly better (*P*<.001) than the corresponding performance scores of supervised learning (Table 1, ADS-sds vs TargetOnly). When using 1000 target-domain training examples, the best ADS system, ADS-fsa, achieved 0.790 AUC-ROC and 0.850 average precision, also significantly better (*P*<.001) than that achieved by supervised learning (Table 1, ADS-fsa vs TargetOnly).

Our evaluation set was challenging, because terms included in this set had been prefiltered (ie, ranked as high) by 2 term-ranking methods (details in the Training and Evaluation Datasets subsection). In other words, we evaluated ADS on a set of candidate terms that had higher quality than the average candidate terms mined from EHRs, for which the boundaries between positive and negative examples were more subtle. For example, some candidate terms (eg, “metastatic carcinoid tumor,” “normal serum calcium,” and “acute cardiac ischemia”), although registered as medical terms in UMLS, were judged nonimportant or nonurgent for lay definition creation because their meanings could be easily inferred from their component words.

The evaluation results on this dataset suggest that our ADS system is effective in ranking EHR terms and can be used to facilitate the expansion of lexical resources that support EHR comprehension. In particular, it can be used to alleviate the data sparseness problem when there are very few target-domain training data and can be used to boost the performance of supervised learning when the size of the training data increases.

### Effects of Target-Domain Training Data

Our evaluation results also suggested that using more target-domain training data is beneficial for system performance (rows 2-4 in Table 1). In an additional experiment (details in Multimedia Appendix 4), we found that the performance of ADS-fsa, the best system when using 1000 target training data, continued to improve with increased target training data and began to plateau when the number of target training examples reached 2500.

### Effects of Individual Features

The results of our feature ablation experiment (Table 2) indicate that word embedding contributes mostly to system performance, followed by general-domain term frequency and term length. Although dropping semantic features had mixed effects, the results from further analysis indicate that semantic features are useful when excluding word embedding from the feature set. Specifically, adding semantic features on the 3 other types of features (ie, automatic term recognition, general-domain term frequency, and term length) significantly improved system performance (*t*_99_ ranges from 12.74 to 128.11, *P*<.001 for 2 measures under 4 conditions; see the second table in Multimedia Appendix 3 for details). This suggests that most information provided by the semantic features for ranking terms is subsumed by that provided by word embedding (but not vice versa). Different from the semantic features, the automatic term recognition features had little additional effect on the performance even without counting word embedding. A likely reason is that our evaluation data set was created by including terms already ranked as high (top 5%) by the automatic term recognition algorithm C-value [65], which may have diminished the effect of this type of feature on this dataset.

### Comparing Different Transfer Learning Methods

Although ADS-fsa and ADS-sds were both effective in ranking EHR terms (Table 1), ADS-fsa had small gains over ADS-sds when the size of target training data was large (1000 examples) and vice versa when the size of the target training data was small (100 and 200 examples). The 2 systems used different methods, SDS and FSA, to balance the source- and target-domain training data. Specifically, SDS allows fine-grained weighting of training data from source and target domains at the instance level; FSA, by using an augmented feature space, allows redistribution of feature weights for source, target, and “shared” domains. Our results suggest that instance weighting (ie, ADS-sds) can be more effective when the target-domain training data are very limited.

### Error Analysis

We identified three major types of errors through error analysis on the top-rank and low-rank terms (using 300 as the rank threshold) that were ranked by the ADS-sds system that used 1000 target-domain training examples for transfer learning. Error analysis for ADS-fsa showed similar results. First, we found that most errors were caused by compound terms. Specifically, ADS-sds ranked some terms (such as “malignant cell,” “chronic rhinitis,” and “viral bronchitis”) as high, even though their meanings could be easily inferred from their component words. It also ranked certain good compound terms (eg, “community-acquired pneumonia,” “end-stage kidney failure,” and “left ventricular ejection fraction”) as low when these terms contained familiar words. This suggests that advanced features generated by a compound term detector may improve the system’s performance, which we may explore in the future. Second, ADS-sds missed certain terms that are lay terms in the general domain but bear unfamiliar clinical meanings (eg, “baseline,” “vehicle,” and “family history”). Third, ADS-sds ranked some common medical terms (eg, “aspirin,” “vitamin,” and “nerve”) as high, although these terms are likely to be already known by the average patient. The second and third types of errors may be reduced by including domain-specific knowledge about term familiarity as additional features, which we will study in the future.

### Conclusion

We report a novel ADS system for ranking and identifying medical terms important for patient EHR comprehension. We empirically show that the ADS system outperforms strong baselines, including supervised learning, and transfer learning can effectively boost its performance even with only 100 target-domain training examples. The EHR terms prioritized by our model have been used to expand a comprehensive lay language lexical resource that supports patient EHR comprehension. The top 10,000 EHR terms ranked by ADS are available upon request.

## Appendix

Multimedia Appendix 1 Analysis results of consumer health vocabulary’s coverage of terms in electronic health record notes.

Multimedia Appendix 2 Criteria used for manual selection of terms important for patient comprehension of electronic health record notes.

Multimedia Appendix 3 Effects of features on performance of adapted distant supervision.

Multimedia Appendix 4 Effects of increasing target-domain training data on system performance.

## Abbreviations

ADS: adapted distant supervision
AUC-ROC: area under the receiver operating characteristic curve
CHV: consumer health vocabulary
EHR: electronic health record
FSA: feature space augmentation
JATE: Java Automatic Term Extraction
NLP: natural language processing
SDS: supervised distant supervision
UMLS: Unified Medical Language System
