# Supplementary material for: Ranking Medical Terms to Support Expansion of Lay Language Resources for Patient Comprehension of Electronic Health Record Notes: Adapted Distant Supervision Approach
Source: JMIR Med Inform. 2017 Oct 31;5(4):e42. doi: 10.2196/medinform.8531 (PMC5686421; doi:10.2196/medinform.8531)
Supplement: Multimedia Appendix 1 [file medinform_v5i4e42_app1.pdf]

Multimedia Appendix 1. Analysis Results of Consumer Health Vocabulary's Coverage on Terms in Electronic Health Record Notes

In this analysis, we used the Unified Medical Language System (UMLS) lexical tool MetaMap [1] to identify 19,503 unique terms from a corpus of 7,839 electronic health record (EHR) notes and searched these terms in the Consumer Health Vocabulary (CHV) [2]. Among these terms, 4,680 (24.0%) terms do not appear in CHV, including "focal motor deficit", "Hartmann procedure", "titrate", and "urethrorectal fistula", for example. In addition, there are 19,674 medical terms that exist in CHV but do not have lay terms, including "laryngeal carcinoma", "lymphangiomatosis", "neurocytoma", and "nutcracker esophagus", for example. CHV provides lay definitions for 851 (4.3%) of these terms and leaves 18,823 (95.7%) terms without lay definitions. Here, we followed Zeng-Treitler et al. [3] (i.e., CHV familiarity score $\leqslant$ 0.6) to identify medical terms in CHV.

References:

1. Aronson AR, Lang F-M. An overview of MetaMap: historical perspective and recent advances. J Am Med Inform Assoc JAMIA 2010 Jun;17(3):229–236. PMID:20442139

2. Zeng QT, Tse T. Exploring and developing consumer health vocabularies. J Am Med Inform Assoc JAMIA 2006 Feb;13(1):24–29. PMID:16221948

3. Zeng-Treitler Q, Goryachev S, Kim H, Keselman A, Rosendale D. Making texts in electronic health records comprehensible to consumers: a prototype translator. Proc AMIA Annu Symp 2007;846–850. PMID:18693956
