# Supplementary material for: Ranking Medical Terms to Support Expansion of Lay Language Resources for Patient Comprehension of Electronic Health Record Notes: Adapted Distant Supervision Approach
Source: JMIR Med Inform. 2017 Oct 31;5(4):e42. doi: 10.2196/medinform.8531 (PMC5686421; doi:10.2196/medinform.8531)
Supplement: Multimedia Appendix 2 [file medinform_v5i4e42_app2.pdf]

Multimedia Appendix 2. Criteria Used for Manual Selection of Terms Important for Patient Comprehension of Electronic Health Record Notes

Table A2-1. Criteria for judging the importance of candidate terms extracted from *EHR-Pittsburgh*[a]. Positive examples refer to the medical terms important for patient EHR comprehension and negative examples refer to the other terms.

Criteria	Description	Positive examples	Negative Examples
**unithood**	It is a valid term	adrenal insufficiency, iron deficiency anemia, white blood count, VQ scan, femoral nerve block	mouth daily, procedure performed, deficiency anemia, four hour, doctor wash incision
**termhood**	It is a medical term with clear clinical meaning(s) in its standalone form	ACE inhibitor, heart failure, bronchoscopy, esophageal motility study, extravasation	activity, advice, failure, rate, level
**quality of compound term**	Its meaning is beyond the simple sum of the meanings of its component words or sub-terms	acute myeloid leukemia, focal necrosis, Cushing's disease, sinus pause, sixth cranial nerve	acute pancreatitis, alcohol intoxication, head CT, left internal carotid artery, small bowel movement
**unfamiliarity**	It is unfamiliar to the average person	neurocytoma, lymphangiomatosis, laryngeal carcinoma, premalignant, hand-foot syndrome	vitamin, heart disease, tablet, fatigue, infant

[a] 7,839 discharge summary notes (5.4 million words) from the University of Pittsburgh Natural Language Processing Repository (Chapman W.). Using this data requires a license.

Except unithood which is a general criterion for lexical entry selection, the other three criteria all measure term importance from the perspective of patient EHR comprehension. Specifically, termhood measures whether a term has a clear clinical meaning in its standalone form. Clinical terms are often used to describe patients' medical conditions, diagnostic procedures, and treatments in EHRs, and are therefore important for patients to know to comprehend their EHR notes. Term unfamiliarity measures whether a term is unfamiliar to an average person. Defining familiar terms will not help patients much with their EHR comprehension because

they are already known by the average patient. The quality of a compound term affects its importance in two ways. First, high-quality compound medical terms (e.g., "community-acquired pneumonia") should be annotated with lay definitions because otherwise patients would not understand them even if they know all the individual words contained in these terms. Second, creating lay definitions for low-quality compound terms is unnecessary if we have defined the individual words in these terms. By excluding low-quality compound terms, we can speed up the annotation process to define more terms important for patient EHR comprehension within a designated time frame.

When judging term importance, the domain experts checked the termhood and unfamiliarity for single-word terms and checked all the four conditions for multi-word terms. They checked a candidate term's unithood by using linguistic knowledge. *N*-grams (i.e., concatenation of *n* words) that are not valid noun phrases were filtered out. They checked a term's domain relevance (i.e., termhood) and unfamiliarity by using domain knowledge. For example, a medical term that has a lay-language synonym is likely to be unfamiliar to the average person. CHV familiarity scores were also used as a reference. They judged the quality of a compound term based on whether its meaning is decomposable. Information from the internet, e.g., whether a term has lexical entries in multiple authentic health education websites, was also used as a reference.
