# Supplementary material for: Ranking Medical Terms to Support Expansion of Lay Language Resources for Patient Comprehension of Electronic Health Record Notes: Adapted Distant Supervision Approach
Source: JMIR Med Inform. 2017 Oct 31;5(4):e42. doi: 10.2196/medinform.8531 (PMC5686421; doi:10.2196/medinform.8531)
Supplement: Multimedia Appendix 3 [file medinform_v5i4e42_app3.pdf]

Multimedia Appendix 3. Effects of Features on Performance of Adapted Distant Supervision

Table A3-1. Performance of different ADS-fsa[a] systems implemented by using all types of features or by respectively dropping each individual type of features, under 4 conditions using 100, 200, 500, and 1000 target-domain training examples[b].

ADS-fsa system	AUC-ROC[c]				Average Precision			
	100	200	500	1000	100	200	500	1000
ADS-fsa-ALL[d]	0.746	0.756	0.776	0.790	0.815	0.823	0.839	0.850
ADS-fsa-woWE[e]	0.702 *t*=30.50 *P*<.001	0.711 *t*=33.01 *P*<.001	0.721 *t*=64.88 *P*<.001	0.730 *t*=125.98, *P*<.001	0.772 *t*=25.66 *P*<.001	0.778 *t*=33.36 *P*<.001	0.788 *t*=59.58 *P*<.001	0.795 *t*=105.42, *P*<.001
ADS-fsa-woSem[f]	0.749 *t*=2.69 *P*=.01	0.756	0.774 *t*=3.32 *P*=.001	0.785 *t*=13.82 *P*<.001	0.821 *t*=4.98 *P*<.001	0.826 *t*=2.97 *P*=.004	0.840	0.848 *t*=5.24 *P*<.001
ADS-fsa-woATR[g]	0.746	0.756	0.776	0.790	0.816	0.823	0.839	0.850
ADS-fsa-woTF[h]	0.735 *t*=10.72 *P*<.001	0.746 *t*=11.98 *P*<.001	0.767 *t*=15.59 *P*<.001	0.781 *t*=27.86 *P*<.001	0.809 *t*=5.69 *P*<.001	0.818 *t*=6.69 *P*<.001	0.835 *t*=8.06 *P*<.001	0.845 *t*=14.68 *P*<.001
ADS-fsa-woTL[i]	0.736 *t*=10.67 *P*<.001	0.748 *t*=8.61 *P*<.001	0.768 *t*=17.79 *P*<.001	0.782 *t*=40.83 *P*<.001	0.803 *t*=15.54 *P*<.001	0.813 *t*=12.42 *P*<.001	0.830 *t*=19.97 *P*<.001	0.842 *t*=43.03 *P*<.001

[a]ADS-fsa: adapted distant supervision-feature space augmentation.
[b]We report the *P* values (if the *P* value ≤.05) and the corresponding t_{99} values for differences between each implementation and ADS-fsa-ALL.
[c]AUC-ROC: area under the receiver operating characteristic curve.
[d]ADS-fsa-ALL: ADS-fsa with all types of features.
[e]ADS-fsa-woWE: ADS-fsa without word embedding.
[f]ADS-fsa-woSem: ADS-fsa without semantic features.
[g]ADS-fsa-woATR: ADS-fsa without features derived from automatic term recognition.
[h]ADS-fsa-woGTF: ADS-fsa without general-domain term frequency.
[i]ADS-fsa-woTL: ADS-fsa without term length.

Table A3-2. Performances of different ADS[a] systems implemented by using baseline features derived from automatic term recognition, general-domain term frequency, and term length or by adding semantic features to the baseline features, under 4 conditions using 100, 200, 500, and 1000 target-domain training examples[b].

ADS system	AUC-ROC[c]				Average Precision			
	100	200	500	1000	100	200	500	1000
ADS-fsa-base[d]	0.642	0.650	0.664	0.669	0.660	0.685	0.730	0.744
ADS-fsa-base +Sem[e]	0.702	0.711	0.721	0.730	0.772	0.778	0.788	0.795
t_{99} and *P* values (ADS-fsa-base+Sem vs. ADS-fsa-base)	*t*=32.26 *P*<.001	*t*=35.11 *P*<.001	*t*=41.38 *P*<.001	*t*=75.90 *P*<.001	*t*=31.86 *P*<.001	*t*=20.07 *P*<.001	*t*=14.58 *P*<.001	*t*=25.56 *P*<.001
ADS-sds-base[f]	0.657	0.665	0.669	0.671	0.713	0.734	0.743	0.747
ADS-sds-base +Sem[g]	0.711	0.718	0.726	0.733	0.780	0.785	0.793	0.799
t_{99} and *P* values (ADS-sds-base+Sem vs. ADS-sds-base)	*t*=21.38 *P*<.001	*t*=33.92 *P*<.001	*t*=58.62 *P*<.001	*t*=128.11 *P*<.001	*t*=12.74 *P*<.001	*t*=13.06 *P*<.001	*t*=20.33 *P*<.001	*t*=83.51 *P*<.001

[a]ADS: adapted distant supervision.
[b]We report the *P* values and the corresponding t_{99} values between ADS-fsa-base and ADS-fsa-base+Sem and between ADS-sds-base and ADS-sds-base+Sem.
[c]AUC-ROC: area under the receiver operating characteristic curve.
[d]ADS-fsa-base: ADS-feature space augmentation using baseline features derived from automatic term recognition, general-domain term frequency, and term length
[e]ADS-fsa-base+Sem: ADS-feature space augmentation using baseline features plus semantic features.
[f]ADS-sds-base: ADS-supervised distant supervision using baseline features derived from automatic term recognition, general-domain term frequency, and term length.
[g]ADS-sds-base+Sem: ADS-supervised distant supervision using baseline features plus semantic features.
