# Supplementary material for: Ranking Medical Terms to Support Expansion of Lay Language Resources for Patient Comprehension of Electronic Health Record Notes: Adapted Distant Supervision Approach
Source: JMIR Med Inform. 2017 Oct 31;5(4):e42. doi: 10.2196/medinform.8531 (PMC5686421; doi:10.2196/medinform.8531)
Supplement: Multimedia Appendix 4 [file medinform_v5i4e42_app4.pdf]

Multimedia Appendix 4. Effects of Increasing Target-domain Training Data on System Performance

In this additional experiment, we investigated whether the performance of adapted distant supervision-feature space augmentation (ADS-fsa) would plateau when using larger target training set (L>1000). We randomly selected 4000 examples from the 6038 annotated terms as the target-domain training set and used the remaining 2038 terms as the evaluation set. We used the same procedure as described in the subsection "Target-domain Training and Evaluation Sets" to create 100 pairs of <target training set, evaluation set> and reported system performance by using L (L=100, 200, 500, 1000, 1500, 2000, 2500, 3000, 3500, 4000) examples randomly selected from the full target training set. In this experiment, we only evaluated the ADS-fsa system which performed best when using 1000 target training examples and used the TargetOnly system as the baseline.

Figure A4-1 shows the performance trend of the ADS-fsa system and the TargetOnly system when increasing the target-domain training examples from 100 to 4000 (see Table A4-1 for the value of each data point). Specifically, the performance scores of both systems improved with increased target training data and the improvements began to plateau when the number of target training examples reached 2500. In addition, ADS-fsa performed better than TargetOnly consistently at all conditions.

Figure A4-1. Effects of increasing target-domain training data on performance of adapted distant supervision-feature space augmentation (ADS-fsa), using TargetOnly as a baseline. N is the number of target-domain training examples. AUC-ROC: area under the receiver operating characteristic curve.

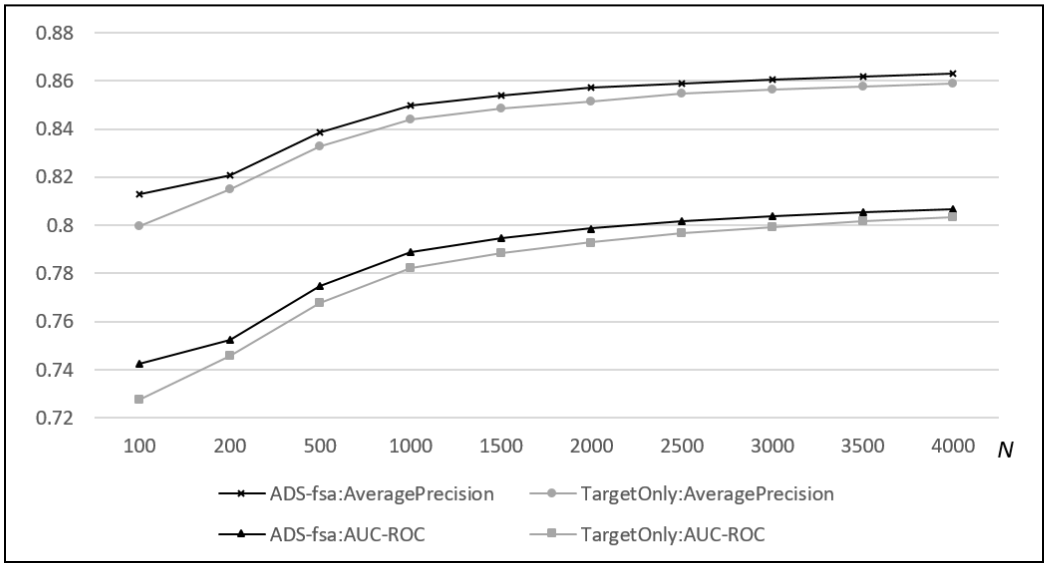

Table A4-1. Effects of increasing target-domain training data on system performance of ADS-fsa[a], using TargetOnly as a baseline[b].

	AUC-ROC[d]			Average Precision		
N[c]	TargetOnly	ADS-fsa	t_{99} and P values	TargetOnly	ADS-fsa	t_{99} and P values
100	0.728	0.743	t=10.53 P<.001	0.780	0.813	t=10.02 P<.001
200	0.746	0.752	t=6.25 P<.001	0.815	0.821	t=6.72 P<.001
500	0.768	0.775	t=10.17 P<.001	0.833	0.839	t=8.91 P<.001
1000	0.782	0.789	t=17.34 P<.001	0.844	0.850	t=12.09 P<.001
1500	0.789	0.795	t=18.66 P<.001	0.849	0.854	t=12.75 P<.001
2000	0.793	0.799	t=19.78 P<.001	0.852	0.857	t=15.02 P<.001
2500	0.797	0.802	t=19.03 P<.001	0.855	0.859	t=12.56 P<.001
3000	0.799	0.804	t=20.03 P<.001	0.856	0.861	t=14.56 P<.001
3500	0.802	0.805	t=18.45 P<.001	0.858	0.862	t=14.49 P<.001
4000	0.803	0.807	t=16.47 P<.001	0.859	0.863	t=16.81 P<.001

[a]ADS-fsa: adapted distant supervision-feature space augmentation.
[b]We report the P values and the corresponding t_{99} values between ADS-fsa and TargetOnly.
[c]N: the number of target-domain training examples.
[d]AUC-ROC: area under the receiver operating characteristic curve.
